# Contributions to the History of Development of the Teeth

**Published:** 1888-03

**Authors:** Carl Heitzmann, C. F. W. Bödecker


### Independent Practitioner.

VOL. IX. MARCH, 1888. NO. 3.

NOTE.—No paper published or to be published in another journal will be accepted for this department. All papers must be in the hands of the Editor before the first day of the month preceding that in which they are expected to appear. Extra copies will be furnished to each contributor of an accepted original article, and reprints, in pamphlet form, may be had at the cost of the paper, press-work and binding, if ordered when the manuscript is forwarded. The Editor and Publishers are not responsible for the opinions expressed by contributors. The journal is issued promptly, on the first day of each month.

### Original Communications.

### CONTRIBUTIONS TO THE HISTORY OF DEVELOPMENT OF THE TEETH.

BY CARL HEITZMANN, M. D., AND C. F. W. BÖDECKER, D. D. S., M. D. S.

CONTINUED FROM PAGE 66.

M. Pfluger (Deutsche Vierteljarsschrift für Zahnheilkunde, 1867, page 167) gives a complete and concise record of the earliest literature of our subject, and we offer a translation of that portion:

"Eustach, in 1574, made the first discovery of the germs of the incisors and eye-teeth, but did not find those of the back teeth. He also stated that he found the tooth-sacs (Zahnfächer) filled with a substance appearing like plaster of Paris, by which the teeth were driven out of their sockets. After this time nothing was done till Leuwenhoek, in 1722, again made some microscopical examinations. In 1728 the development of the teeth was described by Fauchard, who stated that they grew in layers from inward to the periphery, and that the temporary teeth were provided with roots.

"In 1745 Herissant first noticed the development of the separate tissues of the tooth. He believed that the enamel was formed by glands which were present upon the inner surface of the tooth-sac. In 1771 van Swieten and Brunner asserted that the temporary teeth were rootless, but that if they were left in the mouth too long, they might sprout roots. In 1780 Robert Blake, in his *Diss. Inaug.*, stated that the teeth were covered by cement, or crusta petrosa, which was shown by Flourens to be present on the teeth of animals, and later, Alexander Nasmyth discovered the presence of this substance upon human teeth.

"The first to give any of the details of the development of the teeth was Delabarre, in 1815, who stated that previous to the eruption of the teeth he found no openings in the gums which would represent the apertures of the sacs in which the germs of the teeth are situated, and through which they afterward grow. Serres, in 1817, very correctly described the time of the formation of the tooth-germs. Delabarre gave the result of his investigation in 1819, as follows:

1. "The periosteum of the alveolus is a special membrane for the socket.

2. "The membranes of the tooth are, through the periosteum, in connection with very many nerves, arteries and lymphatic fibers.

3. "On every tooth-germ there is present an appendix of different size.

4. "The tooth-sacs communicate with openings upon the gums.

"In 1830 Leszai published a rather creditable work, which, however, was incomplete in regard to the temporary teeth. This author described a horny substance, and mentions the presence of two membranes in a tooth sac."

Pflüger then, after quoting the more recent writers, enters upon the origin of the epithelial cord. He could easily trace the origin of the spindle epithelium (cylindrical), but was at first unable to account for the presence of the round cells between these two layers, till very patient studies convinced him that they originated from the cylindrical epithelium (*spindelförmigen Zellen*). He describes the development of the epithelial cord into the cup shape, which is filled by the dentinal papilla. He noticed that during the process of development the external epithelium, by lateral expansion, becomes less distinct, which this author thinks might be produced by the pressure occasioned through the metamorphosis of the round cells into star-shaped ones. He also noticed the formation of the tooth-sac, as well as that of the enamel, which latter, he states, is formed by the cells which lie close upon the dentine, but he does not state in what manner. He again calls attention to the stellate reticulum, which, as this author expresses it, looks very much like connective tissue, and which is gradually lost as the development of the enamel advances.

A. Retzius (Arch. f. Anat. Physiol. and Wiss. Med. [Müller's], 1837, pages 486 to 565) discusses thin sections of teeth obtained while yet in their alveoli, by means of saws and files. He preferred fresh specimens, on account of their elasticity during their preparation. He observed that the enamel was composed of six-sided prisms, which exhibited cross lines (Striæ of Retzius), giving it the appearance of being composed of little blocks. He was not able to give an explanation of their origin, but thought that every little block of enamel was enveloped by organic substance, and thus the striated appearance was produced.

This author describes a membrane thought to be present between the layer of enamel and dentine in fully formed teeth. He states that the enamel prisms have one end resting upon this membrane, while their distal extremity ends upon the periphery of the enamel. Retzius observed the stratification, as well as the pigmentation, of the enamel, and believes the former originates from the different periods of calcification during the formation of the enamel. The pigmentation he believes to arise either from stoppages in the process of the development of the enamel, or a grouping together of the transverse lines (Striæ of Retzius) in the enamel prisms, or from both causes. He also states that Leuwenhoek (1678) had described these lines in the teeth.

Retzius in his resumé (page 563) compares the structure of the enamel to that of the crystalline lens, and believes that the enamel, like the former, is nourished by the fluids of the blood through the dentinal canals, which then, by means of the thin films (Hautwände), such as the author states to be present around each little enamel block, establish a circulation in the enamel. He devotes a great deal of space to a description of the dentinal canaliculi, their size in different localities, their general course and divisions. He believes that the canaliculi have separate walls, and are filled with an organic earthy material. The cementum he believes to be identical with bone tissue, and states that it ends as a very thin layer on the neck of the tooth. Besides describing human teeth, this author gives a good account of the teeth of twenty-six different animals.

John Goodsir (Edinburgh Medical and Surgical Journal, January, 1839,) gives a clear description of the macroscopical appearances, together with the primitive dental groove of the jaws of fœtus, as well as the formation of the papillæ, which he first noticed about the second month of intra-uterine life. These papillæ (as he describes them) gradually become surrounded by a ridge, which grows up from the mucous membrane of the gum until at last it forms a perfect enclosure around the papilla, this at the same time sinking down into the body of the jaw. He also describes the formation of the lateral laminæ which enclose the sacs transversely, and later on mentions the enamel organ, in which he found no vessels. He also observed the follicles of the permanent teeth, which he describes as at first situated above the sacs of the temporary teeth, but afterward sinking to their sides. This author divides the development of the teeth into stages; the follicular, the saccular, and the eruptive, and one preceding these, the papillary stage.

J. Henle (Allgemeine Anatomie, Leipzig, 1841, page 859,) observed the stratification as well as pigmentation of the enamel. The former he believes to arise from the wavy course of the enamel prisms. As regards the origin of pigmentation he agrees with Purkinje, who believes it to arise from the cutting of the section through bundles of wavy enamel prisms, which when cut in this position are thought to refract the light in a peculiar way. He states (page 861) that Serres discovered in the gums of the fœtus and new born children some gland-like granules (evidently the nests and buds of the external epethelium, which the writers have depicted in figs. 5 and 6), and which Serres called *Glandulæ Tartaricæ,* being of opinion that they secrete the calculary deposits, but found these formations to contain thin epethelial plates, and believes them to be ordinary mucous follicles.

Henle states that already Herissant, in 1754, had described small openings upon the gums of embryos which communicated with the tooth sacs, and through which the teeth were shed, and after quoting Goodsir's publication regarding the formation of the papillæ, he states that the inner surface of the enamel organ (Zahnsäckchens) is smooth and lies upon the dentine germ, the surface of which is covered by the membrana præformativa. The cells are lengthened and stand in a regular row, but higher up he found round and other varieties of cells, which during the growth of the dentine arrange themselves and become cylindrical cells, and these form the fibers and their branches.

Henle further states that the enamel organ envelops the dentine papillæ like a cup, the inner layer of which is lined by a row of cylindrical or polygonal cells, arising in the same manner as those of the dentinal papillæ (odontoblasts). This author observed a film between the enamel organ and the formed enamel, and on page 868 he says: "At first the uppermost layer of (enamel) fibers are in contiguity with the enamel organ, gradually separating itself from it and becoming an independent film, which may be called the enamel film, or membrana adamantina." He observed no vessels in the enamel organ. He further states that the dentine contains open tubes which are filled with a fluid. He believes that the cement may be formed partially by the tooth sac.

Robert Blake (An Essay on the Structure and Formation of the Teeth of Man and Various Animals, Baltimore, 1848,) observed about the fourth month the rudiments of vascular membranes of twelve teeth in each jaw, all the temporary as well as the first permanent molars. He found these to be intimately connected with the gums, and thus believed that they were derivations from it. He also noticed the enamel organ in which he observed no blood-vessels. The enamel he calls "cortex striatus," and regards it as a substance merely composed of crystals, and formed by a membrane, which, after having performed its peculiar function, is totally wasted or absorbed. He further states that a part of this membrane remains on the body of the tooth, which, however, is destroyed when the tooth has risen to its proper height. Blake is of opinion that the cortex striatus (enamel), when the tooth penetrates the gums, is as hard and perfect as it can be at any future period of life, and does not after that period receive the slightest degree of nutrition. He also observed anomalous formation of the enamel, which he attributes to constitutional derangements during its formation. He frequently observed on otherwise healthy human teeth some parts of the enamel to be quite soft, while in the different classes of the lower animals that he examined he never saw a single instance in which the enamel was imperfect. On page 84 he quotes a statement from Wooffendale, who attributes defects of the enamel to small-pox, and when present on both the temporary and permanent teeth Wooffendale suspects that such a child must have had the small-pox twice.

R. B. Todd and W. Bowman (Anatomy and Physiology of Man, Philadelphia, 1850, page 532) regard the teeth on account of their origin from the oral mucosa as tegumentary appendages. They observed that the primitive dental groove was formed about the sixth week, being later transformed into the enamel organ, and that this forms the lining of the follicle and is reflected over the surface of the papilla. They also mention the stellate reticulum (enamel pulp) which originates between the two layers of epithelium, and is separated from the dentinal pulp by only a row of columnar epithelium (the enamel matrix), while, as they state, "on the opposite surface the blood-vessels of the membrane lining the alveolus are seen coming up to, and forming loops immediately under, the enamel pulp, without penetrating it." It is further remarkable that short tubes filled with glandular epithelium descend among these vessels from the enamel pulp, and end with blind extremities. How these tubes (the knobs and buds of the external epithelium as described in figs. 5 and 6), which are evidently glandular, can discharge their contents, it is diffcult to understand, seeing that they appear to open into the substance of the enamel pulp. These authors express the possibility that the stellate reticulum between the two rows of epithelia only performs the mechanical duty of protecting the growing enamel from injury, and provide space for development. They also observed that prior to the calcification of the dentine the nucleated particles of the pulp arrange themselves in rows, and multiply by transverse divisions. The origin of the basis substance of the dentine they explain in such a manner that the basis substance and the tubes originate from the cell itself, and the dentinal fibers are formed from their respective nuclei, which elongate and coalesce, the process being, as they state, similar to the formation of bone tissue. The formation of the enamel they believe to be that the enamel cells (ameloblasts) undergo direct calcification, and that other cells are added when the first have been used up in calcification until the enamel has obtained its proper thickness. They state, "It is from that surface of the enamel pulp which looks toward the tooth that this successive development of new enamel columns proceeds. As they form, this tissue wastes, but it is not probable that the pulp is converted into the columns as the dentinal pulp is converted into dentine, because the anatomical characters of the pulp are so dissimilar from those of the columns."

L. S. Beale (On the Structure and Growth of the Tissues and on Life, London, 1865, page 148), in the formation of dentine, observed between the formed dentine and the vascular pulp a layer which looked like nucleated columnar epithelium, and further says: "Although the pulp diminishes in size while the formation of the dentine proceeds, the pulp does not become the dentine. * * * This dentine results from changes occurring in a tissue which lies upon the surface of the pulp. This consists of cell-like columnar tissue. * * * As new dentine is formed, these cells encroach upon the pulp, the constituent tissues of which gradually diminish in amount." The author agrees with J. Tomes that the dentinal canals are not empty, but contain living fibrils, which he was able to stain with carmine. He also states that the dentinal fibers are formed from the nuclei (germinal matter) of the cells, while the basis-substance of the dentine represents his formed material, which substance calcifies in the form of small globules, gradually increasing in size, and often several of these coalesce. The formation of enamel, he believes, is accomplished in about the same manner as that of the dentine, but he is opposed to calling the layer of enamel cells membrana præformativa, yet states that the membrane raised by the action of acetic acid consists of the altered outer uncalcified part of the columnar cells, which he believes to be Nasmyth's membrane. He further says: "There are numerous enamel cells (in describing a specimen) which are calcified in the lower part, near the dentine, while the more superficial portion remains granular, and still contains a large nucleus. The enamel cell increases in length as the so-called nucleus moves away in a direction from the dentine." In the formation of the cementum, Beale is of the opinion that this tissue is not the result of an ossification of the tooth-sac, but that it is formed by cell action identical to the formation of bone tissue. On page 172

Beale enters into the discussion of the homology of the dentine, enamel and cementum, and says: "I look upon both enamel and dentine as calcified 'epithelial structures;'" coming to this conclusion through the fact that dentine has a greater resemblance to epithelium than bone; also, that hairs, like teeth, grow from follicles.

A very interesting case of healed fracture of a tooth, with a new formation of enamel, is found in Virchow's Archiv. (Bd. 38, Heft. 4), by H. Hertz. The tooth was extracted by Brücke from the mouth of a servant girl eighteen years of age, who at the age of eight had a fall, when the tooth, a left upper lateral, was fractured. The crown of the tooth, after this accident, was directed outward. Hertz describes the probable healing process of the dentine and cementum, but is unable to explain the new formation of the enamel which was observed upon this tooth. He also published an article in Deutsche Vierteljahrsschrift für Zahnheilkunde, 1870, page 181, on tooth fractures, in which he gave the history of fourteen healed fractures of teeth.

E. F. Wenzel, who wrote an extensive article on researches about the enamel organ and the enamel of animals (Archiv der Heilkunde, 1868, page 97), in describing the enamel cells of a sheep, says (page 101) that sometimes he observed a light rim on that part of the enamel cells which lies toward the enamel, but that he was unable to observe anything like the membrana præformativa described by Huxley to be present between the enamel cells and the enamel prisms, for the purpose of excreting the enamel.

Carl Wedl (The Pathology of the Teeth, Philadelphia, 1872), regarding the formation of the enamel, holds the opinion that it is produced by direct calcification of the ameloblasts (enamel cells) into the enamel. In regard to the enamel organ, he observed that with the growth of the tooth it becomes thinner, and finally shrivels up completely. His views concerning the formation of dentine are that "as soon as the connection of the dentinal with their formative cells occurs, the calcification of the dentinal cells begins; their principal and accessory processes remain attached and are transformed into dentinal fibers." Wedl is convinced that the odontoblasts are the only formations capable of producing dentine, and when this occurs in places where no odontoblasts are present, it can only be done by an inversion of the odontoblast layer (membrana eboris). The occurrence of the globular territories in the dentine he explains as the result of a coalescence of separate groups of calcified dentinal cells. On the formation of the cementum he says: "At the margin of the crown the dental sac contracts, and upon its inner surface the formation of the cement is effected, increasing gradually as the formation of the root advances; the lower segment of the dental sac becomes, therefore, the root membrane of the tooth." On the structure of Nasmyth's membrane he agrees with J. Tomes, viz., that it belongs to the cement.

(TO BE CONTINUED.)

